# Variation of butyrate production in the gut microbiome in type 2 diabetes patients

**DOI:** 10.1007/s10123-023-00324-6

**Published:** 2023-02-13

**Authors:** Julienne Siptroth, Olga Moskalenko, Carsten Krumbiegel, Jörg Ackermann, Ina Koch, Heike Pospisil

**Affiliations:** 1 High Performance Computing in Life Sciences, Technical University of Applied Sciences Wildau, Wildau, Germany; 2 BIOMES NGS GmbH, Schwartzkopffstraße 1, 15745 Wildau, Germany; 3 Department of Molecular Bioinformatics, Institute of Computer Science, Goethe University Frankfurt, 60325 Frankfurt am Main, Germany

**Keywords:** Type 2 diabetes, Gut, Butyrate, NGS, Statistical analysis

## Abstract

**Background:**

Diabetes mellitus type 2 is a common disease that poses a challenge to the healthcare system. The disease is very often diagnosed late. A better understanding of the relationship between the gut microbiome and type 2 diabetes can support early detection and form an approach for therapies. Microbiome analysis offers a potential opportunity to find markers for this disease. Next-generation sequencing methods can be used to identify the bacteria present in the stool sample and to generate a microbiome profile through an analysis pipeline. Statistical analysis, e.g., using Student’s t-test, allows the identification of significant differences. The investigations are not only focused on single bacteria, but on the determination of a comprehensive profile. Also, the consideration of the functional microbiome is included in the analyses. The dataset is not from a clinical survey, but very extensive.

**Results:**

By examining 946 microbiome profiles of diabetes mellitus type 2 sufferers (272) and healthy control persons (674), a large number of significant genera (25) are revealed. It is possible to identify a large profile for type 2 diabetes disease. Furthermore, it is shown that the diversity of bacteria per taxonomic level in the group of persons with diabetes mellitus type 2 is significantly reduced compared to a healthy control group. In addition, six pathways are determined to be significant for type 2 diabetes describing the fermentation to butyrate. These parameters tend to have high potential for disease detection.

**Conclusions:**

With this investigation of the gut microbiome of persons with diabetes type 2 disease, we present significant bacteria and pathways characteristic of this disease.

## Background

Diabetes mellitus is one of the most prevalent diseases worldwide. In 2019, the World Health Organization (WHO) included diabetes mellitus in the top 10 leading causes of death (WHO 2020). Diabetes mellitus is a metabolic disease, with some hereditary predispositions. It is divided into two main variants, type 1 and type 2. Type 2 diabetes mellitus (T2D) is the most common form and accounts for over 90% of all cases. In this disease, the pancreas still provides sufficient insulin, but this can be increasingly poorly processed by the cells until a complete insulin resistance is developed (Zaccardi et al. 2016). Lack of exercise, diet, and obesity are regarded risk factors (Fletcher et al. 2002). In the past, this disease was considered to be a consequence of old age, but nowadays this disease is also increasingly occurring in children and youth. Diabetes mellitus type 2 disease can lead to the development of cardiovascular diseases, diabetic foot and damage to the kidneys and eyes, and can even lead to death (Cannon et al. 2018). Studies have shown that the disease can be pushed back or even defeated completely by changing the way of life (Lean et al. 2018).

The intestinal microbiota is a complex structure of bacteria, fungi and virus. Bacteria account for the largest share, with a total of about 100 trillion bacteria living in human intestines. In addition to digestion, the intestine is involved in many other processes and is also called the control center of the body. For example, it supports the immune system and controls inflammatory processes, among other things. The intestinal microbiome is not only influenced by nutrition, but also by many other factors, such as lifestyle, environment, age and gender. Thus, the composition of the intestinal microbiota varies depending on these factors. With a balanced diet, the diversity of bacteria in the intestine increases, and this leads to a wide formation of various metabolic products (Lozupone et al. 2012; Shreiner et al. 2015). In this context, the short-chain fatty acids (SCFA) are of particular importance (Valdes et al. 2018). An important representative is butyrate, which is produced by a variety of bacteria. Butyrate producers make up about 20% of the total bacterial community. Most frequently occurring representatives are members of the families *Lachnospiraceae* and *Ruminococcaceae* (Vital et al. 2017). Butyrate is formed during the fermentation of carbohydrates via various pathways (pyruvate, succinate, etc.) (Vital et al. 2014). In this process, it represents one of the most important sources of energy for intestinal cells. In addition, butyrate is considered to have a health-promoting effect. It not only ensures the functionality of intestinal cells, but also strengthens the intestinal barrier, regulates immune functions and controls metabolic processes. A reduction of butyrate-producing bacteria is associated with the development of diseases (Mallott and Amato 2022).

With the advent of research into the microbiome, links between gut bacteria and type 2 diabetes mellitus have also emerged. Although the question remains whether causality can be inferred between the correlations found. However, increased research in the field is leading to a better understanding. Existing correlations can be confirmed and deepened, and new ones can form the basis for further studies. Thus, consequences and causes of diseases and changes in the intestinal microbiome can be better understood and possible therapeutic approaches can be developed (Arora and Tremaroli 2021; Li et al. 2020; Sharma and Tripathi 2019; Gurung et al. 2020).

## Materials and methods

### Data

The available data sets consist of microbiome and individual lifestyle data. These are not from a clinical survey with medical supervision, but are extensive due to the low-threshold manner of the survey. More than 29,000 samples were available, each associated with individual lifestyle information. The data (only with indication of consent for scientific use) is provided by the company BIOMES NGS GmbH as a project partner within the scope of its business-like activity. BIOMES NGS GmbH offers a self-test for the analysis of the intestinal flora. Both the sampling and the answering of the questions about the individual lifestyle were performed by the customers themselves. It is a lifestyle product without any further verification of the customer’s information by a medical doctor.

The data on individual lifestyle includes information on age, center of life, height and body weight, as well as information on diet, diseases and medication intake. A total of 99 fields were covered.

The microbiome profile was composed of normalized counts per taxonomic level (kingdom, phylum, class, order, family, genus, species) from sequenced bacterial 16S ribosomal DNA (rDNA). Microbial DNA was analyzed using next-generation sequencing (NGS). This has advantages over classical gut analysis methods because the analysis is more accurate and thus the entirety of the intestinal microbiome can be determined. The 16S rDNA, a gene of the microorganisms, is analyzed.

One subset consisted of clients, who have a diabetes type 2 disease with an age between 18 and 80.

A total of 272 samples met the inclusion criteria, of which 143 were female and 127 were male, and two samples had no gender information.

The diabetes type 2 subgroup was compared with a healthy reference group. Samples for the healthy reference were selected based on the listed parameters.

Inclusion criteria (healthy):

- Age 18-80
- No diseases
- No gastrointestinal complains
- No allergies gluten intolerance
- No medication
- No antibiotic intake < 3 months
- No probiotic intake < 3 months
- BMI 18.5 to 27.5
- Alcohol intake not daily
- Good wellbeing (> = 5) & health score (>= 6 out of 10)

These criteria resulted in a group size of 674 healthy individuals. Of these, 340 were female, 318 were male, 1 are socially diverse, and 15 samples without gender information. Table 1 shows the distribution of the selected parameters age, gender, BMI, nutrition and sports for the two groups Healthy and T2D. There was no significant difference in nutrition between the two groups. The T2D group did slightly less sports than the Healthy group. This is consistent for the development of diabetes mellitus type 2 disease. For the investigated data set, further adjustment of the parameters in the two groups was not possible, as otherwise unacceptable group sizes would have resulted. The parameters age and BMI are known risk factors and crucial for the development of diabetes type 2. Thus, higher age and higher BMI are characteristic.

**Table 1:** Distribution of the selected parameters age, gender, BMI, nutrition and sports for the two groups Healthy and T2D

Parameter	Healthy group (n = 674)	T2D group (n = 272)
Age, years	42,55 ± 12,12	59,71 ± 12,27
Women/Men/Divers, n	340/318/1	143/127/0
BMI, *kg*/*m*^2^	23,13 ± 2,24	31.05 ± 6.38
Nutrition		
Omnivore	517	245
Vegan	40	2
Vegetarian	74	10
Pescetarian	43	8
Sports		
Daily	22	3
5–6 times per week	61	5
3–4 times per week	199	31
1–2 times per week	172	28
1 time per week	52	17
1 time per 2 weeks	18	7
Less 2 months	2	5
Never	132	107

## Methods

### Sample preparation and sequencing

#### Sample storage and lysis

Collected stool samples are stored in 1000 μL DNA-stabilizing buffer at − 20 ^∘^C until use. For the lysis process, the samples are defrosted and will be centrifuged. Afterwards, warmed up PW buffer from the QIAamp 96 PowerFecal QIAcube HT Kit is added to each sample.

#### Extraction of stool samples

For the extraction we established the QIAamp 96 PowerFecal QIAcube HT Kit on our liquid handling systems (Hamilton StarLine & Tecan EVO) by using a vacuum chamber as well as a high-pressure chamber. The extracted gDNA is stored at − 20 ^∘^C until use.

#### Library preparation for sequencing with the Illumina MiSeq System

The library preparation follows the manual “16S Metagenomic Sequencing Library Preparation- Preparing 16S Ribosomal RNA Gene Amplicons for the Illumina MiSeq System”. The mastermix reagents for the target and library amplification are from New England BioLabs, 16S V3V4 primer from Eurofins. For normalization of all samples, a fluorescent dye, and the Biotek Synergy HTX plate reader are used to measure DNA concentrations and to calculate the necessary dilution volume per sample. To ensure a high throughput, all the steps, from the first amplification to the library pooling, are nearly fully automated by using the liquid handling systems (Hamilton StarLine). Hence, we can process between 96 and 192 samples simultaneously and the normalization also works for up to 288 samples. The Library Denaturing and MiSeq Sample Loading is carried out manually.

### Processing sequence reads

The determined paired-end reads were filtered in the following. First, the forward/reverse reads were merged using PANDAseq (Masella et al. 2012). This was followed by an alignment using BLASTn (Altschul et al. 1990) against the SILVA rRNA database (version: 138.1) (Quast et al. 2013). Afterwards, filtering was performed. There must be at least 10,000 assigned reads for each sample, for further analysis. Different identity thresholds per taxonomic boundaries (phylum: 75.0%, class: 78.5%, order: 82.0%, family: 86.5%, genus: 94.5%, species: 97.0%) (Yarza et al. 2014) were used. The sequences were clustered according to their similarity (97%) using CD-HIT (Li and Godzik 2006; Fu et al. 2012). Biologically normalized abundances were calculated from the clustered reference sequences using the PICRUSt2 pipeline (Douglas et al. 2020). The output is a table of biologically normalized counts per taxonomic level. The PICRUSt2 pipeline was used to determine the available pathways (MetaCyc Caspi et al. 2016) for each sample applying a predictive model. The abundances of the identified pathways were calculated based on the gene families.

The alpha diversity measures Shannon entropy (Shannon 1948) and inverse Simpson correlation (Simpson 1949) were determined from the rarefied raw counts. The mathematically normalized values were calculated using QIIME2 (Bolyen et al. 2019). Shannon entropy is a measure of diversity and includes both the number of different species and the number of individuals per species. The Shannon index originated in information theory, as a measure of the information content of a message. The inverse Simpson index is also a measurement for describing diversity and, like the Shannon index, belongs to the alpha diversity measures. It is based on the Simpson index. This indicates the probability that two randomly selected individuals belong to different species. The inverse Simpson index is the reciprocal of the Simpson index.Further analysis steps were performed with custom Python scripts using the pandas (McKinney 2010; The pandas development team 2020), NumPy (Harris et al. 2020), scikit-learn (Pedregosa et al. 2011) and SciPy (Virtanen et al. 2020) libraries.

### Statistical analysis

Various statistical tests were applied to detect significant differences between the two groups T2D and Healthy. To determine whether the numerical values of microbiome and lifestyle data follow a normal distribution, a test based on D’Agostino and Pearson (D’Agostino 1971; D’Agostino and Pearson 1973) was used. A Student’s t-test (Student 1908) was applied to normally distributed samples, and a Mann-Whitney U test (Wilcoxon 1945) was applied to non-normally distributed samples. To determine correlations of individual lifestyle categorical data, the Chi-Square Test of Independence was used. A p-value of > 0.05 was considered significant for all tests used.

## Results

### Diversity of the intestinal microbiome

To identify which bacteria/taxa are significant to each group, diversity was examined first. This was done by calculating the number of bacteria per taxonomic level that had at least one relative count. The quantity of different bacteria per subgroup for each taxonomic level is listed in Table 2. Occurrences over the entire group were also included. It can be seen that the values in the T2D group were about 10% lower than in the Healthy control group across all taxonomic levels.

**Table 2:** Diversity: number of bacteria (at least one relative count) per taxonomic level for the groups Healthy and T2D and in the total of both groups

Group	Phylum	Class	Order	Family	Genus	Species
Total	75	224	490	1045	2393	4326
Healthy	75	217	460	966	2140	3690
T2D	67	184	409	874	1911	3275

**Fig. 1:**
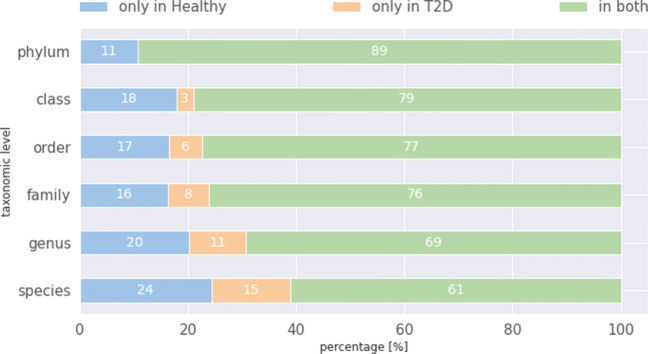
Ratio of bacteria present only in the Healthy group (blue), those present only in the T2D group (orange), and in both groups (green), in percent for each taxonomic level

The uniqueness of bacteria in those groups was investigated afterwards. These percentage values are shown in Fig. 1. The majority of bacteria occur in both groups. In addition, more bacteria occur only in the group Healthy. To determine whether the unique bacteria are characteristic for diabetes, they were tested for significance. For this purpose, different hypothesis tests were applied, Student’s t-test for normally distributed samples and Mann-Whitney U test for non-normally distributed samples. A p-value of > 0.05 was assumed as significant. None of the unique bacteria were found to be characteristic of either group.

A hypothesis test was performed for bacteria present in both groups to check for significance. For normally distributed samples, a Student’s t-test was applied, and Mann-Whitney U test was used for samples not following a Gaussian distribution. A p-value of 0.05 was assumed as significance threshold. Of 2393 different genera, 25 were identified as significant. Of these, the count values for 4 genera (*Bacteroides*, *Blautia*, *Lachnoclostridium*, and *Prevotella*) were increased in the T2D group in comparison to the Healthy group. The other 19 genera (*Faecalibacterium*, *Lachnospira*, *Roseburia*, *Ruminococcus*, etc.) showed decreased count values in T2D compared to Healthy. Bacteria that could not be precisely assigned (non-specific, etc.) were not included. Table 3 lists significant bacteria (genus level) with respecting p-value and relative abundance in the T2D group.

**Table 3:** Bacteria at genus level determined to be significant, with p-value and inclusion of the T2D group relative to the Healthy group (*⇑* — increases; *⇓* — decreases)

Genus	Occurrence in diabetes group	p-value
*Lachnoclostridium*	*⇑*	7.751e-22
*Bacteroides*	*⇑*	1.308e-05
*Blautia*	*⇑*	2.169e-03
*Prevotella*	*⇑*	1.644e-03*
*Lachnospiraceae FCS020 group*	*⇓*	2.460e-22
*Lachnospiraceae ND3007 group*	*⇓*	6.416e-14
*Faecalibacterium*	*⇓*	8.775e-12
*Ruminococcus*	*⇓*	7.398e-11
*Clostridium sensu stricto 1*	*⇓*	3.000e-10*
*Lachnospiraceae UCG-001*	*⇓*	2.885e-08
*Coprococcus*	*⇓*	5.091e-07
*Subdoligranulum*	*⇓*	4.141e-06
*Lachnospira*	*⇓*	3.639e-06
*Lachnospiraceae NC2004 group*	*⇓*	2.348e-06
*Lachnospiraceae NK4A136 group*	*⇓*	2.284e-06
*Fusicatenibacter*	*⇓*	4.077e-05
*Lachnospiraceae UCG-006*	*⇓*	2.310e-05
*UCG-002*	*⇓*	7.378e-03
*Roseburia*	*⇓*	5.050e-03
*Marvinbryantia*	*⇓*	3.677e-03
*Lachnospiraceae UCG-008*	*⇓*	2.881e-03
*Agathobacter*	*⇓*	2.438e-03*
*Alistipes*	*⇓*	2.560e-02*
*Butyricicoccus*	*⇓*	1.747e-02*
*Anaerostipes*	*⇓*	1.288e-02

*Mann-Whitney U test

The significant genera belong to 9 different families (out of 1045 different families). Thereby, the family of *Lachnospiracaeae* was the most represented one (16 genera out of 25), followed by *Ruminococcacaeae* (3 out of 25). For the remaining 6 genera, each belongs to different families.

### Quantification by alpha diversity

**Fig. 2:**
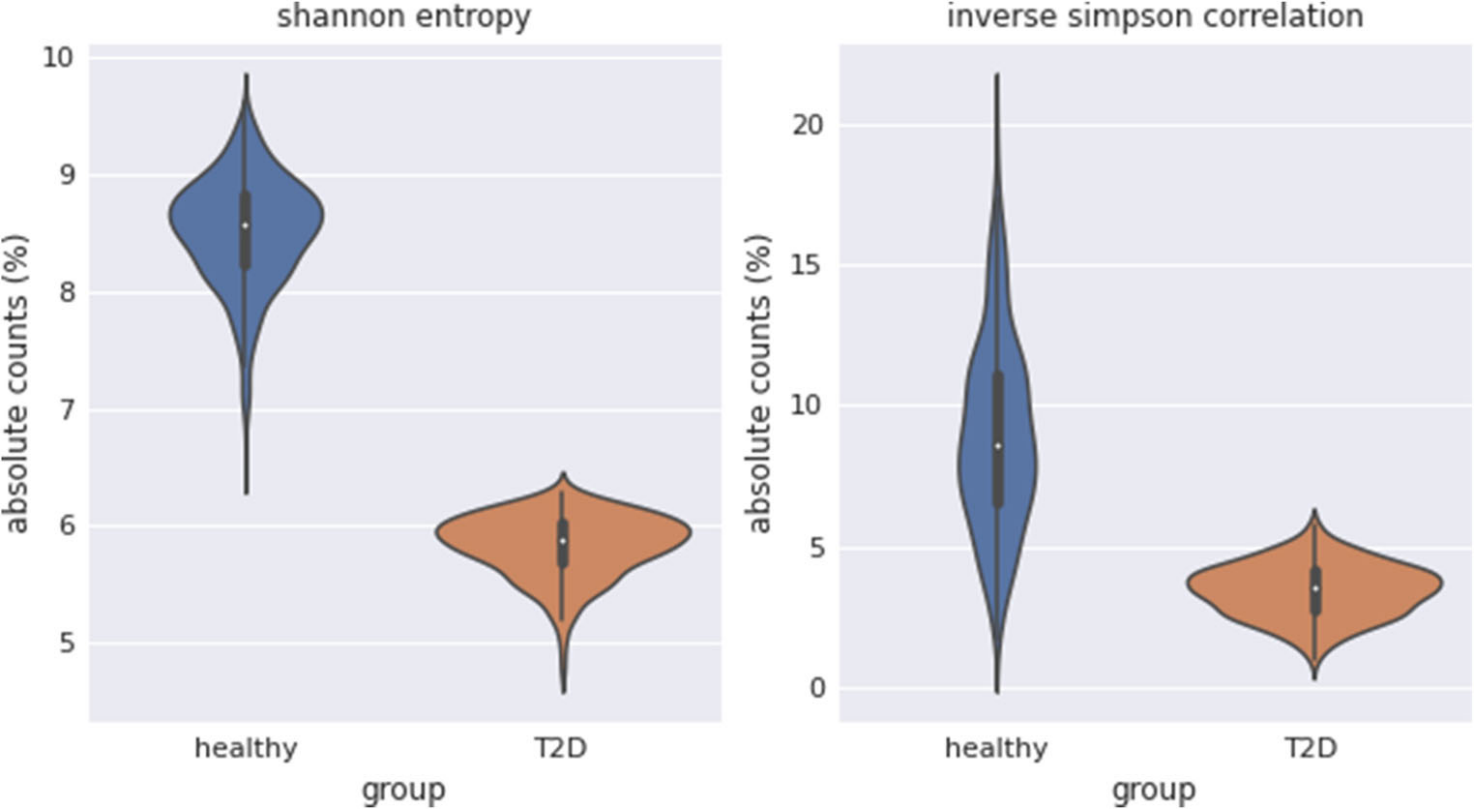
Alpha diversity: violin plot for the group Healthy (blue) and the group T2D (orange) of absolute counts (%) for Shannon entropy (left) and inverse Simpson correlation (right)

To describe the diversity of the groups, the Shannon entropy and the inverse Simpson correlation were calculated. Figure 2 shows the results as a violin plot for both groups. Both parameters for alpha diversity are significantly increased in the Healthy group compared to the T2D group. This is particularly visible for the Shannon entropy.

### Functional microbiome: consideration of the butyrate production pathways

Furthermore, the identified pathways were analyzed for their significance for type 2 diabetes mellitus. Special attention was paid to pathways in which butyrates are produced. The significant pathways are listed in Table 4. These are six pathways in which fermentation to butanoate occurs. High significance was found for three of six pathways (*PWY-5677*, *P163-PWY*, *CENTFERM-PWY*; cf. Table 4). The three other pathways (*P162-PWY*, *PWY-5676*, *PWY-5022*) showed weak significance.

**Table 4:** Pathways with butyrate as end product

BioCyc ID	Pathways	p-value
*PWY-5677*	Succinate fermentation to butanoate	1.570e-12
*P163-PWY*	l-Lysine fermentation to acetate and butanoate	4.804e-10
*CENTFERM-PWY*	Pyruvate fermentation to butanoate	2.312e-05
*P162-PWY*	l-Glutamate degradation V (via hydroxyglutarate)	6.015e-02
*PWY-5676*	Acetyl-CoA fermentation to butanoate	8.599e-02
*PWY-5022*	4-Aminobutanoate degradation V	2.278e-01

## Discussion

This work analyzed the intestinal microbiome of 272 individuals with diabetes mellitus type 2 disease and 674 healthy control subjects. Significant differences in the alpha diversity measures, Shannon entropy and inverse Simpson correlation, were found between the two groups. It became clear that the diversity in the diabetes mellitus type 2 group is lower than in the Healthy control group. This was also confirmed by other studies, which show that the microbiome diversity of persons suffering a disease is lower compared to healthy persons (Zhang et al. 2019; Larsen et al. 2010).

Furthermore, the analyses demonstrated that there are bacteria that are only present in one of the two comparison groups. However, the occurrences of these bacteria were not significant for diabetes mellitus type 2 disease. Characteristic bacteria for distinguishing the diabetes microbiome from healthy microbiome were present in both groups. The only significant difference between the genera was in the amount of their occurrence. Four genera (*Bacteroides*, *Blautia*, *Lachnoclostridium* and *Prevotella*) showed an increased occurrence in the diabetes mellitus type 2 group than in the Healthy control group. The other significant genera (*Anaerostipes*, *Coprococcus*, *Fusicatenibacter*, *Lachnospira*, *Marvinbryantia*, *Roseburia*, *Faecalibacterium*, *Ruminococcus*, *Subdoligranulum*, *UCG-002*, *Agathobacter*, *Butyricicoccus*, *Alistipes*, *Clostridium sensu stricto 1* and all *Lachnospiraceae*) showed the opposite.

The genus *Blautia* was one of the most represented genera in both groups enriched in group T2D. Previous studies confirm this behavior (Egshatyan et al. 2015; Zhang et al. 2013).

*Lachnoclostridium* were more abundant in the microbiome profiles of diabetic patients. This association has not yet been verified in other studies. However, there are studies in relation to other diseases (Kang et al. 2021). Further research is necessary to identify *Lachnoclostridium* as a marker for type 2 diabetes mellitus. There are anomalies in the genera *Bacteroides* and *Prevotella*, both of which are increased in the T2D group, compared to the healthy group. Although similar behavior was detected in other studies, both genera are propionate producers. Propionate, like butyrate, belongs to the short-chain fatty acids. These are associated with a healthy status of the microbiome (Wu et al. 2010; Candela et al. 2016).

A significant reduction of genera *Alistipes*, *Anaerostipes*, *Ruminococcus* was detected in the T2D group. This decrease was associated with diabetes mellitus type 2 and was supported by previous studies. *Ruminococcus* and *Anaerostipes* were associated with a healthy state of the microbiome and showed increased values in the Healthy control group (Gao et al. 2018; Doumatey et al. 2020). Furthermore, various studies also indicated a decrease in genera in the group T2D (Zhang et al. 2019; Gaike et al. 2020; Liu et al. 2020; Salamon et al. 2018; Das et al. 2021).

A decrease of *Alistipes* counts was also identified in the data of group T2D (Thingholm et al. 2019).

Additionally, the analysis pointed out a reduction in the *Lachnospira*, *Roseburia*, *Faecalibacterium* and *Coprococcus* genera in the T2D group compared to the healthy group. These genera were butyrate producers and were associated with healthy gut flora. The decrease of these genera favored obesity and the development of diseases. This fact and other studies supported the assumption that these were genera associated with diabetes mellitus type 2 disease. In these studies, a reduction was also detected in the T2D group (Zhang et al. 2019; Larsen et al. 2010; Kang et al. 2021; Candela et al. 2016; Gao et al. 2018; Liu et al. 2020; Salamon et al. 2018; Das et al. 2021; Anand et al. 2016).

The genus *Subdoligranulum* is closely related to the genera *Faecalibacterium*, both from the family *Ruminococcaceae*. Like *Faecalibacterium*, *Subdoligranulum* is also a butyrate producer. Furthermore, this genus was associated with a healthy metabolic status, but the exact physiological role is yet unknown (Van Hul et al. 2020). The results (cf. Table 3) show that the occurrence of *Subdoligranulum* is reduced in the group with type 2 diabetes mellitus. The reduction of *Subdoligranulum* can also be assumed to have a negative impact and is thus characteristic of type 2 diabetes mellitus disease.

In four other genera (*Fusicatenibacter*, *Agathobacter*, *Butyricicoccus*, *Marvinbryantia*), a decrease in the T2D group could also be detected. Those are currently not associated with type 2 diabetes mellitus. All genera produce short-chain fatty acids, e.g., butyrates, and are associated with a healthy intestinal flora (Kang et al. 2021; Lu et al. 2019; Ma et al. 2020; Chen et al. 2021). The reduction of genera leads to a lower proportion of short-chain fatty acids in the organism. This favors obesity and the development of diseases. Thus, these genera may be elements of a profile for the detection of type 2 diabetes mellitus. The exact relationship between the genera *Fusicatenibacter*, *Agathobacter*, *Butyricicoccus* and *Marvinbryantia* and type 2 diabetes mellitus disease also needs to be investigated in further trials.

The consideration of the functional microbiome has received greater attention in recent years. It is assumed that not only individual bacteria/taxa, but also their interactions are decisive. So characteristic profiles, e.g., diseases and lifestyle, can be found. Thus, pathway analyses are becoming more and more important. In this work, pathways were investigated in which fermentation to butanoate takes place (Qin et al. 2012; Reichardt et al. 2014). Six different pathways (*PWY-5677*, *P163-PWY*, *CENTFERM-PWY*, *P162-PWY*, *PWY-5676*, *PWY-5022*) could be determined. Through significance analysis, these were examined with respect to their relevance for the group T2D. Furthermore, significant, determined taxa (e.g., *Lachnospira*, *Roseburia*, *Faecalibacterium*, and *Coprococcus*) are also identified as butyrate producers. These facts support the assumption that the functional microbiome has a high importance for the analysis of the microbiome. Further research must verify the potential found for associations with diabetes mellitus type 2 disease or even other diseases or lifestyles. For this purpose, additional pathways need to be examined. These may also be approaches for early detection and therapies (Arora and Tremaroli 2021).

## Data Availability

The data used are not publicly available because the research project was carried out in collaboration with the company BIOMES NGS GmbH. They can be requested with a reasoned request to the corresponding author.

## Abbreviations

BMI: body mass index
NGS: next-generation sequencing
rDNA: ribosomal deoxyribonucleic acid
SCFA: short-chain fatty acids
T2D: type 2 diabetes
WHO: World Health Organization
